# Relationships Among Cognitive Distortions, Forgiveness of Others, and Self-Forgiveness in Psychiatric Outpatients with Depressive Disorders: A Cross-Sectional Study

**DOI:** 10.3390/healthcare14111478

**Published:** 2026-05-27

**Authors:** Selin Saklamaz, Havva Akpınar

**Affiliations:** 1 Department of Psychiatric Nursing, Institute of Health Sciences, Mugla Sıtkı Kocman University, Mugla 48000, Türkiye; selinozcelik34@gmail.com; 2 Department of Psychiatric Nursing, Faculty of Health Sciences, Mugla Sıtkı Kocman University, Mugla 48000, Türkiye

**Keywords:** depressive disorder, cognitive distortions, forgiveness of others, self-forgiveness, psychiatric outpatients

## Abstract

**Highlights:**

**What are the main findings?**

- Cognitive distortions were negatively associated with forgiveness of others in psychiatric outpatients with depressive disorders.
- A weak positive relationship was identified between forgiveness of others and self-forgiveness, whereas no significant relationship was found between cognitive distortions and self-forgiveness.

**What are the implications of the main findings?**

- The findings suggest that forgiveness of others and self-forgiveness may be differently associated with cognitive distortions in patients with depressive disorders.
- Evaluating cognitive distortions together with forgiveness-related processes may contribute to a more comprehensive psychosocial assessment in depressive disorders.

**Abstract:**

**Background**: Depression is associated with cognitive, emotional, and interpersonal difficulties that may influence forgiveness-related processes. This study aimed to examine the relationships among cognitive distortions, forgiveness of others, and self-forgiveness in patients with depression. **Methods**: This cross-sectional and correlational study was conducted with 252 voluntary psychiatric outpatients diagnosed with selected ICD-10 depressive disorders in a single outpatient clinic, excluding individuals with severe depressive episodes and psychotic features. Data were collected using the Descriptive Data Form, the Rye Forgiveness Scale (RFS), the State Self-Forgiveness Scale (SSFS), and the Cognitive Distortions Scale (CDS). Descriptive statistics, independent-samples t-tests, one-way analysis of variance (ANOVA), Pearson correlation analyses, and linear regression analyses were used for data analysis. **Results**: A weak positive correlation was found between forgiveness of others and self-forgiveness (r = 0.206, *p* < 0.001), whereas a moderate negative correlation was identified between forgiveness of others and cognitive distortions (r = −0.486, *p* < 0.001). No statistically significant relationship was found between cognitive distortions and self-forgiveness (*p* > 0.05). Regression analysis showed that cognitive distortions significantly predicted forgiveness of others. Female participants had significantly higher self-forgiveness scores than males, and primary school graduates had higher cognitive distortion scores than high school and university graduates. **Conclusions**: The findings suggest that cognitive distortions may be more strongly associated with forgiveness of others than with self-forgiveness in psychiatric outpatients with depressive disorders. Further longitudinal and intervention-based studies are needed to better clarify these relationships.

## 1. Introduction

Depression is one of the most common mental health disorders and significantly affects individuals’ functioning, interpersonal relationships, and quality of life. It may lead to important individual and societal consequences, including recurrent episodes, loss of workforce, psychosocial dysfunction, and an increased risk of suicide [1]. In the World Health Organization’s (WHO) Mental Health Gap Action Programme (mhGAP), depression is recognized as a priority mental health condition, and the importance of psychosocial interventions for individuals with depression is emphasized [2].

According to Beck’s cognitive theory, depression is explained within the framework of cognitive processes related to cognitive schemas, cognitive distortions, the cognitive triad, and automatic thoughts. Cognitive distortions refer to dysfunctional, unrealistic, and negatively biased patterns of thinking regarding the self, the environment, and the future. These distortions, which emerge as a result of negative cognitive schemas, may contribute to the development and maintenance of depressive symptoms by influencing individuals’ interpretations of events [3]. Previous studies have reported positive associations between cognitive distortions and levels of depression and anxiety, and cognitive distortions have also been associated with depressive symptoms, hopelessness, rumination, and interpersonal problems [4,5].

Forgiveness is defined as a psychological process associated with individuals’ ability to cope with feelings of hurt, anger, and other negative emotions, move away from thoughts of revenge, and develop empathy, understanding, and positive emotions [6,7,8,9]. Self-forgiveness, on the other hand, is a more subjective process involving individuals’ ability to cope with their own mistakes, guilt, and feelings of regret. Previous studies have reported that forgiveness and self-forgiveness are positively associated with psychological well-being, life satisfaction, and psychological resilience, whereas they are negatively associated with depressive symptoms, stress, and negative emotions [10,11,12,13,14,15,16,17].

Several theoretical models have been proposed to explain forgiveness. Enright’s process model conceptualizes forgiveness as a cognitive and emotional process consisting of the stages of uncovering, decision, work, and deepening [18]. The REACH Forgiveness model developed by Worthington conceptualizes forgiveness as a structured process involving recalling the hurt, developing empathy, viewing forgiveness as an altruistic act, committing to forgiveness, and maintaining forgiveness over time [19]. Both models emphasize that forgiveness is associated with cognitive appraisal, emotional regulation, and interpersonal processes.

Previous studies have reported a strong positive association between forgiveness and mental health [20]. In addition, forgiveness tendency has been associated with a lower likelihood of depression [21], and forgiveness has been reported to positively influence individuals’ psychosocial well-being while reducing psychological distress [22]. Furthermore, a study conducted by Zhang et al. (2020) found that the presence of depressive symptoms was associated with lower levels of forgiveness among individuals who had been hurt by others [23].

A positive relationship between cognitive distortion levels and depressive symptoms has also been reported among individuals diagnosed with depression. In addition, cognitive distortions have been associated with anxiety symptoms, adult separation anxiety, social anxiety, negative self-perception, hopelessness, rumination, aggression, dysphoria, and suicide attempts. Moreover, reductions in cognitive distortions have been associated with decreases in depressive symptoms [4,5,24].

Cognitive distortions may influence how individuals evaluate interpersonal events, which may in turn be associated with forgiveness and self-forgiveness processes. In particular, the frequent presence of negative cognitive evaluations, guilt, anger, rumination, and interpersonal conflicts among individuals with depression suggests that forgiveness-related processes may be associated with cognitive structures. Nevertheless, the existing literature examining cognitive distortions, forgiveness of others, and self-forgiveness together remains limited. In particular, studies evaluating these variables simultaneously in clinical samples of patients with depression are insufficient. Therefore, the present study aimed to examine the relationships among cognitive distortions, forgiveness of others, and self-forgiveness in patients with depression.

The study sought to answer the following research questions:

1. What are the levels of forgiveness of others, self-forgiveness, and cognitive distortions in patients with depression?
2. Do forgiveness of others, self-forgiveness, and cognitive distortions differ according to participants’ sociodemographic characteristics?
3. Is there a relationship among forgiveness of others, self-forgiveness, and cognitive distortions in patients with depression?

Based on the relevant literature, the following hypotheses were established:

**H1.**  *Cognitive distortions are significantly associated with forgiveness of others in patients with depression.*

**H2.**  *Cognitive distortions are significantly associated with self-forgiveness in patients with depression.*

**H3.**  *Forgiveness of others is significantly associated with self-forgiveness in patients with depression.*

**H4.**  *Forgiveness of others, self-forgiveness, and cognitive distortions differ according to participants’ sociodemographic characteristics.*

## 2. Materials and Methods

### 2.1. Study Design

This study employed a cross-sectional and correlational design to examine the relationships among cognitive distortions, forgiveness, and self-forgiveness in patients with depression. The study was conducted and reported in accordance with the Strengthening the Reporting of Observational Studies in Epidemiology (STROBE) guidelines [25]. Due to the cross-sectional nature of the study, causal relationships, directionality among variables, and intervention-related effects cannot be determined. Therefore, the findings should be interpreted as associations rather than evidence of causation.

### 2.2. Participants

The target population of the study consisted of individuals diagnosed with depressive disorders who attended the psychiatry outpatient clinic of a training and research hospital. The annual number of patients admitted to the outpatient clinic with World Health Organization’s International Statistical Classification of Diseases and Related Health Problems, 10th Revision (ICD-10; World Health Organization, Geneva, Switzerland) codes related to depressive disorders, including F32 and F33 diagnostic categories, between 1 January 2020, and 31 December 2020, was used as a reference for estimating the accessible population size during the study planning process (N = 12,185). This figure was used only for sample size estimation and did not represent the actual recruitment frame.

The initial sample size was calculated using the formula proposed by Salant and Dillman for finite populations [26]. Based on a 95% confidence level and a ±5% margin of error, the minimum required sample size was calculated as 242 participants In addition, an a priori power analysis was conducted using G*Power version 3.1.9.7 (Heinrich Heine University Düsseldorf, Düsseldorf, Germany) to evaluate the adequacy of the sample size for the planned analyses [27]. Assuming a medium effect size (f^2^ = 0.15), α = 0.05, power = 0.95, and one predictor, the minimum required sample size was calculated as 89 participants. Therefore, the final sample size of 252 participants exceeded both the population-based and power-based minimum sample size requirements.

Participants were recruited using a consecutive sampling approach. During the data collection period, individuals who attended the psychiatry outpatient clinic met the inclusion criteria and agreed to participate were consecutively invited to the study until the required sample size was exceeded. Data were collected between 1 May 2022 and 31 December 2022 in a designated interview room at the psychiatry outpatient clinic. Completion of the forms took approximately 10 min.

#### 2.2.1. Inclusion Criteria for Study Participants

Prior to participant recruitment, diagnoses were verified through hospital medical records. Because the hospital information system used the ICD-10participant eligibility was determined according to ICD-10 diagnostic codes related to depressive disorders [28].

Participants diagnosed with F32 (Depressive episode), F32.0 (Mild depressive episode), F32.1 (Moderate depressive episode), F32.8 (Other depressive episodes), F32.9 (Unspecified depressive episode), F33 (Recurrent depressive disorder), F33.0 (Recurrent depressive disorder with mild current episode), F33.1 (Recurrent depressive disorder with moderate current episode), F33.4 (Recurrent depressive disorder in remission), F33.8 (Other recurrent depressive disorders), and F33.9 (Unspecified recurrent depressive disorder) according to ICD-10 diagnostic criteria [28] were considered eligible for participation.

Participants were included if they:

(1) attended the psychiatry outpatient clinic during the study period;
(2) voluntarily agreed to participate in the study;
(3) were able to read, understand, and complete the questionnaires independently;
(4) completed the study forms.

#### 2.2.2. Exclusion Criteria for Study Participants

Patients diagnosed with severe depressive episodes and psychotic features according to ICD-10 diagnostic criteria [28], including F32.2 (Major depressive episode without psychotic symptoms), F32.3 (Major depressive episode with psychotic symptoms), F33.2 (Recurrent depressive disorder with current major episode without psychotic symptoms), and F33.3 (Recurrent depressive disorder with current major episode with psychotic symptoms), were excluded because these conditions may impair attention, concentration, communication, and the ability to reliably complete self-report measures.

Participants were excluded if they:

(1) had severe depressive episodes or psychotic features according to ICD-10 diagnostic criteria;
(2) had psychiatric diagnoses other than depressive disorders recorded in hospital medical records;
(3) had difficulty understanding or completing the self-report forms; or
(4) submitted incomplete questionnaires.

These criteria were applied to reduce potential confounding effects and to ensure reliable completion of the self-reporting instruments.

### 2.3. Data Collection Instruments

Descriptive Data Form, Rye Forgiveness Scale (RFS), State Self-Forgiveness Scale (SSFS), and “Cognitive Distortions Scale (CDS)” were used.

#### 2.3.1. Descriptive Data Form

The Descriptive Data Form was developed by researchers based on a review of the relevant literature [8,9,15,20], and consisted of 5 items designed to obtain descriptive information regarding participants’ sociodemographic characteristics. The form included questions regarding age, gender, marital status, educational status, and employment status. Diagnostic information based on ICD-10 [28] depressive disorder categories was obtained separately from hospital medical records. Prior to participant recruitment and data collection, medical records were reviewed to verify participants’ diagnoses and determine eligibility for the study.

#### 2.3.2. Rye Forgiveness Scale

The Rye Forgiveness Scale (RFS) was originally developed by Mark S. Rye (1998) and later revised by Rye et al. (2001) [29]. The scale is designed to assess individuals’ levels of forgiveness toward others in situations involving perceived injustice or accusations [29]. The Turkish validity and reliability study of the scale was conducted by Havare and Gizir (2020) [30]. The Turkish version consists of 15 items and three dimensions: negative thoughts and behaviors toward forgiveness, positive thoughts and behaviors toward forgiveness, and emotional responses. However, in the present study, only total scale scores were used in the analyses, and subscale scores were not analyzed separately. The RFS is a 5-point Likert-type scale ranging from “strongly disagree (1)” to “strongly agree (5)”. Items 1, 3, 4, 5, 8, 10, 12, and 14 are reverse scored. RFS example item: “I cannot stop thinking about how unfairly this person treated me.” The scale has no cut-off value, and higher scores indicate a greater tendency to forgive others. Participants completed the scale according to the standardized instructions of the instrument. Specifically, they were instructed to respond while considering individuals who had treated them unfairly or caused them harm. Therefore, the scores obtained in this study reflect forgiveness-related responses within a personally relevant context rather than completely generalized responses. The Cronbach’s alpha coefficient of the Turkish version was reported as α = 0.75 [30]. In the present study, subscale reliability coefficients were also examined. For the RFS, Cronbach’s alpha coefficient for the total scale was α = 0.884. The Cronbach’s alpha coefficients for the subscales were α = 0.876 for negative thoughts and behaviors toward forgiveness, α = 0.879 for positive thoughts and behaviors toward forgiveness, and α = 0.871 for emotional responses.

#### 2.3.3. State Self-Forgiveness Scale

The State Self-Forgiveness Scale (SSFS) was originally developed by Wohl, DeShea, and Wahkinney (2008) to assess individuals’ feelings, attitudes, and beliefs regarding themselves after making a mistake or engaging in perceived wrongdoing [31]. The Turkish validity and reliability study of the scale was conducted by Havare and Gizir (2020) [30]. According to the Turkish validity and reliability study, the scale consists of 12 items and three dimensions: self-forgiveness-related emotions and behaviors, positive self-forgiveness beliefs, and negative self-forgiveness beliefs. However, in the present study, only total scale scores were used for analysis, and subscale scores were not analyzed separately. The SSFS is a 4-point Likert-type scale with responses ranging from “never (1)” to “always (4)”. SSFS example item, “When I think that something I did was wrong, I feel compassion toward myself.” The scale does not have a cut-off score, and total scores range from 12 to 48, with higher scores indicating a greater tendency toward self-forgiveness. Participants completed the scale according to the standardized instructions of the instrument. Specifically, they were instructed to respond while considering situations in which they had done something wrong or believed they had treated someone unfairly. Therefore, the obtained scores reflect self-forgiveness-related responses within a personally relevant context rather than completely generalized responses. The Cronbach’s alpha coefficient for the Turkish version of the scale was reported as α = 0.87 [30]. For the SSFS, the Cronbach’s alpha coefficient for the total scale in the present study was α = 0.85. The coefficients for the subscales were α = 0.84 for self-forgiveness-related emotions and behaviors, α = 0.83 for positive self-forgiveness beliefs, and α = 0.83 for negative self-forgiveness beliefs.

#### 2.3.4. Cognitive Distortions Scale

The Cognitive Distortions Scale (CDS) was developed by Covin et al. (2011) to assess major maladaptive cognitive distortions [32]. The Turkish validity and reliability study was conducted by Ardanıç (2017) [33]. The scale evaluates cognitive distortions related to interpersonal/social situations and personal achievement situations. The cognitive distortions assessed include mind reading, catastrophizing, all-or-nothing thinking, emotional reasoning, labeling, mental filtering, overgeneralization, personalization, should statements, and discounting positive experiences. For example, the “mind reading” dimension assesses the tendency to assume that others hold negative thoughts about the individual despite limited objective evidence, such as believing that another person is dissatisfied or critical despite the absence of explicit negative feedback [33].

The CDS consists of 20 items and is rated on a 7-point Likert-type scale ranging from “never (1)” to “always (7)”. Total scores range from 20 to 140, with higher scores indicating greater cognitive distortion tendencies. Although the scale allows evaluation of total scores and subdomains (interpersonal/social and personal achievement), only total scale scores were used in the present study. The Cronbach’s alpha coefficient reported in the Turkish validity and reliability study was α = 0.88 [33]. In the present study, Cronbach’s alpha coefficients were α = 0.90 for the total scale, α = 0.89 for the interpersonal/social domain, and α = 0.88 for the personal achievement domain.

### 2.4. Data Analysis

Data were analyzed using IBM SPSS Statistics version 25.0 (SPSS Inc., Chicago, IL, USA). Descriptive statistics, including frequencies, percentages, means, and standard deviations, were used to summarize participants’ characteristics and study variables. To assess whether the study variables followed a normal distribution, skewness and kurtosis values were examined. In the present study, skewness values ranged between −0.390 and 0.941, and kurtosis values ranged between −0.625 and 0.332, indicating acceptable normality. According to the relevant literature, skewness and kurtosis values within ±1.5 to ±2.0 are considered indicative of approximately normal distribution [34,35]. Therefore, parametric analyses were performed. Relationships among study variables were examined using Pearson correlation and linear regression analyses. Correlation coefficients (r) were interpreted as follows: 0.00–0.25 very weak; 0.26–0.49 weak; 0.50–0.69 moderate; 0.70–0.89 strong; and 0.90–1.00 very strong [36]. Differences in scale scores according to participants’ descriptive characteristics were examined using independent samples t-tests and one-way analysis of variance (ANOVA). Post hoc comparisons were performed using Tukey’s HSD test where appropriate. Internal consistency reliability was evaluated using Cronbach’s alpha coefficients. Statistical significance was set at *p* < 0.05. Effect sizes were calculated using Cohen’s d and eta-squared (η^2^) coefficients. Cohen’s d values of 0.20, 0.50, and 0.80 were interpreted as small, medium, and large effects, respectively, whereas η^2^ values of 0.01, 0.06, and 0.14 were interpreted as small, medium, and large effects, respectively [34,35].

### 2.5. Ethical Approval

Ethical approval for the study was obtained from the Ethics Committee of a public university (Medical and Health Sciences Ethics Committee-2 (Sports, Health); Date: 23.01.2022, Decision No: 220007-3). Institutional permission was obtained from the hospital where the study was conducted, and permission to use the Turkish versions of the scales was obtained from the corresponding authors responsible for their validity and reliability studies. Participants were recruited on a voluntary basis and informed about the study through an informed consent form. Only individuals who agreed to participate were included in the study. Participants were informed that participation was entirely voluntary and that refusal to participate or withdrawal from the study at any stage would not negatively affect their ongoing treatment or clinical care. To ensure confidentiality and participant privacy, interviews were conducted in a private interview room within the psychiatry outpatient clinic. All procedures were implemented in a standardized manner and scheduled at appropriate times so as not to interfere with routine clinical services, patients’ treatments, or the clinical workflow. Data collection procedures were carried out in accordance with hospital regulations, patient rights, ethical principles, and protective measures. The interview room was located adjacent to the psychiatry examination rooms, allowing immediate access to appropriate clinical support if participants experienced distress or discomfort during the study process. The researcher conducting the data collection was working as a nurse in a psychiatric inpatient unit and was also a graduate student in psychiatric nursing. Therefore, the researcher had professional knowledge and clinical experience regarding therapeutic communication and therapeutic approaches for individuals with depression. Personal information and identifying details were not collected, and all data were handled confidentially. The study was conducted in accordance with the ethical principles of the Declaration of Helsinki and research and publication ethics standards.

## 3. Results

Table 1 presents the descriptive characteristics of patients diagnosed with depression (n = 252). The mean age of the patients was 36.57 ± 12.59 years, with most patients being 30 years younger (36.1%). Slightly more than half of the patients were male (51.2%). Regarding marital status, 47.6% were single. Approximately half of the patients were university graduates (48.0%), and most were employed (60.7%). When the diagnoses of the patients were examined according to the ICD-10 diagnostic classification, the majority showed mild depressive episodes (F32.0) (67.1%), followed by depressive episodes of unspecified subtype (21.0%) and moderate depressive episodes (11.9%).

The participants’ mean scores were 39.78 ± 7.08 for the RFS, 34.20 ± 7.71 for the SSFS, and 78.54 ± 20.98 for the CDS (Table 2).

Differences in scale scores according to patients with depression’s descriptive characteristics are presented in Table 3. A statistically significant difference was found between gender and SSFS scores, with female patients demonstrating higher SSFS scores than male patients (t = −2.519, *p* = 0.012). No significant gender differences were observed for RFS or CDS scores (*p* > 0.05). A significant difference in RFS scores was identified according to marital status (F = 4.412, *p* = 0.013). Post hoc analysis indicated that married patients had significantly higher RFS scores than divorced patients. No statistically significant differences according to marital status were found for SSFS or CDS scores (*p* > 0.05). Educational status was significantly associated with CDS scores (F = 3.319, *p* = 0.021). Post hoc analysis indicated that primary school graduates had significantly higher CDS scores than high school and university graduates. No statistically significant differences in RFS or SSFS scores according to educational status were observed (*p* > 0.05). No statistically significant differences in RFS, SSFS, or CDS scores were found according to ICD-10 diagnostic categories (*p* > 0.05).

Correlations among RFS, SSFS, and CDS scores are shown in Table 4. A weak positive correlation was found between RFS and SSFS scores (r = 0.206, *p* = 0.001). A moderate negative correlation was identified between CDS and RFS scores (r = −0.486, *p* < 0.001). No statistically significant relationship was found between CDS and SSFS scores (r = −0.039, *p* = 0.533).

Linear regression analyses were conducted to examine the effect of CDS scores on RFS and SSFS scores. CDS significantly predicted RFS scores (β = −0.486, *p* < 0.001). The regression model was statistically significant (F = 77.243, *p* < 0.001), explaining 23.3% of the variance in RFS scores (R^2^ = 0.233). In contrast, CDS did not significantly predict SSFS scores (β = −0.039, *p* = 0.533). The corresponding regression model was not statistically significant (F = 0.390, *p* = 0.533), (Table 5).

## 4. Discussion

This study investigated the relationships among forgiveness of others, self-forgiveness, and cognitive distortions in patients with depression. The findings were discussed in line with the relevant literature. Since depression severity was not directly measured using a symptom severity scale, the findings should be interpreted independently from symptom severity levels. Nevertheless, the study sample consisted of patients diagnosed according to ICD-10 criteria with F32 (Depressive episode, unspecified subtype), F32.0 (Mild depressive episode), and F32.1 (Moderate depressive episode) [28].

The mean age of the patients participating in the study was 36.57 ± 12.59 years. Nearly half of the participants were male, university graduates, and single, while more than half were employed and had a diagnosis of mild depressive episode. In a study conducted by Gürsoy (2018) with patients diagnosed with depression, the mean age of the participants was reported as 30.97 ± 10.35 years, and more than half of the participants were single and nearly half were university graduates [37]. These findings are similar to the results of the present study. Although more than half of the patients in our study were employed, Gürsoy (2018) reported lower employment rates among patients with depression [37]. This difference may be related to the fact that the participants in the present study were receiving outpatient treatment, voluntarily agreed to participate in the research, and most had a diagnosis of mild depressive episode.

In the present study, forgiveness of others was evaluated using the RFS, whereas self-forgiveness was assessed using the SSFS. According to the total scale score averages, patients with depression were found to have above-average levels of forgiveness of others and self-forgiveness. Higher RFS and SSFS scores are assumed to reflect greater tendencies toward forgiveness of others and self-forgiveness [30]. In a previous study [6], patients with depression were found to have moderate levels of forgiveness of others and self-forgiveness, and this finding is partially consistent with the results of the present study.

In this study, married participants were found to have higher levels of forgiveness than divorced participants. In a study conducted with women [20], married individuals were reported to have higher forgiveness levels than single individuals, which is consistent with our findings. However, another study conducted with patients with depression [6] found no significant relationship between marital status and forgiveness of others.

According to the findings of the present study, women were found to have higher levels of self-forgiveness than men. A similar finding was reported by Karaca (2019), who found that women had higher self-forgiveness levels and were more likely to forgive themselves than men [38]. However, there are also studies reporting findings different from those of the present study. Several studies [39,40] found no significant gender differences in forgiveness. In contrast, Altay (2022) reported that men were more inclined toward self-forgiveness than women and had more positive beliefs regarding self-forgiveness [41]. Considering the findings of the present study together with the existing literature, further studies are needed to better clarify the relationship between gender and self-forgiveness. Cultural gender roles and differences in emotional processing have been discussed in the previous literature as factors potentially associated with self-forgiveness.

In the present study, cognitive distortion levels of patients with depression were evaluated using the CDS. The mean CDS scores observed in the present study indicated elevated cognitive distortion levels among patients with depression. Higher CDS scores indicate higher levels of cognitive distortions [30]. In a previous study [42], patients with major depression were found to have higher levels of cognitive distortions than healthy individuals, and a positive relationship was identified between depression and cognitive distortion scores. Similarly, other studies conducted with patients diagnosed with depression [5,24,42] reported high levels of cognitive distortions and positive associations between cognitive distortion levels and depression severity. In addition, another study [43], reported a negative relationship between cognitive flexibility and depression. A study conducted with patients diagnosed with major depression also revealed elevated levels of cognitive distortions among patients [44]. Similarly, Gürsoy (2018) found that patients diagnosed with depression and anxiety disorders had higher cognitive distortion levels than healthy control participants [37]. The findings of the present study are consistent with the existing literature. Taken together, these findings support the presence of associations between cognitive distortions and forgiveness-related processes among patients with depression.

In the present study, primary school graduates were found to have higher cognitive distortion levels than high school and university graduates. Similarly, Özkan (2022) reported that cognitive distortion levels decreased as educational level increased [45]. However, some studies [42,46] found no significant relationship between educational status and cognitive distortions. Considering the findings of the present study together with the existing literature, further studies are needed to better clarify the relationship between educational level and cognitive distortions. Lower educational levels may be associated with reduced cognitive flexibility, limited mental health literacy, or fewer adaptive coping resources.

The present study identified a weak positive relationship between forgiveness of others and self-forgiveness among patients with depression. Although the magnitude of this association was limited, the finding is partially consistent with previous studies suggesting that forgiveness of others and self-forgiveness are related but conceptually distinct psychological constructs. Previous research has emphasized that self-forgiveness may involve emotional, cognitive, interpersonal, and moral processes that differ from those involved in forgiving others [7,20]. Similarly, longitudinal findings have shown that forgiveness of self and forgiveness of others may develop through partially independent psychological pathways [47]. In addition, some studies have reported weak or inconsistent associations between different dimensions of forgiveness across clinical and nonclinical populations [48]. In a study conducted by Bankoğlu (2022), a significant negative relationship was identified between forgiveness of others and situational forgiveness [43]. Taken together, these findings suggest that different dimensions of forgiveness may not always be strongly or consistently related to one another. Therefore, the weak correlation observed in the present study may indicate that, although forgiveness of others and self-forgiveness are associated, they should not be considered interchangeable constructs in patients with depression.

In the present study, a moderate negative relationship was identified between forgiveness of others and cognitive distortions among patients with depression. This finding suggests that higher levels of maladaptive cognitive patterns may be associated with lower tendencies to forgive others. Regression analysis further supported this finding, demonstrating that cognitive distortions significantly predicted forgiveness scores and explained 23.3% of the variance in forgiveness. No previous study examining the relationship between forgiveness of others and cognitive distortions in patients with depression could be identified. Forgiveness has been described as an important concept that positively affects both physical and mental health [20]. In another study, a negative relationship was reported between treatment adherence and cognitive distortions among patients with depression who minimized or ignored positive experiences [46]. In addition, Köse and Yıldız (2025), found a positive relationship between psychological resilience and forgiveness [49]. A study conducted with forensic psychiatric patients reported that forgiveness tendencies decreased as alexithymia levels increased [50]. Taken together, these findings suggest that cognitive, emotional, and psychological processes may be associated with forgiveness tendencies.

Self-forgiveness has been reported to increase self-compassion, self-esteem, and emotional well-being while also strengthening individuals’ capacity to cope with psychological stress [49]. Akın and Bilgin (2023) found a low positive relationship between cognitive flexibility and self-oriented perfectionism, whereas a low negative relationship was identified between forgiveness and self-oriented perfectionism [51]. Another study conducted with patients with depression reported a positive relationship between forgiveness and tolerance [20]. In addition, positive associations between psychological well-being and forgiveness levels have also been reported [50]. When these findings are considered together, cognitive distortions, forgiveness, and self-forgiveness may represent related but not entirely overlapping psychological constructs in patients with depression. In the present study, cognitive distortions showed a stronger statistical association with forgiveness of others than with self-forgiveness.

In the present study, no statistically significant relationship was found between cognitive distortions and self-forgiveness. Furthermore, cognitive distortions did not significantly predict self-forgiveness scores in the regression model. No previous study examining the relationship between self-forgiveness and cognitive distortions in patients with depression could be identified. Similarly, Akın and Bilgin (2023) reported no significant relationship between cognitive flexibility and forgiveness [51]. Kaygas and Kılınç (2023) found that hopeless and pessimistic individuals had lower levels of self-forgiveness than hopeful individuals [50]. Likewise, Camadan (2023) reported a positive and significant relationship between self-forgiveness and self-esteem [52]. In a study conducted by Topbaşoğlu Altan and Çivitçi (2020), self-forgiveness and situational forgiveness did not have moderating roles in the relationship between anger and life satisfaction, whereas forgiveness of others showed a moderating role [12].

When the characteristics of self-forgiveness processes and cognitive distortions related to interpersonal relationships are considered together, this finding appears to be consistent with previous literature. Self-forgiveness and cognitive distortions may represent related but conceptually distinct psychological constructs. In the present study, no statistically significant association was observed between cognitive distortions and self-forgiveness. This finding is consistent with the possibility that self-forgiveness processes and interpersonal cognitive distortions may not demonstrate similar patterns of association within this clinical sample [53].

Considering the findings of the present study, several strengths should be acknowledged. First, the study examined the relationships among cognitive distortions, forgiveness of others, and self-forgiveness within a clinical sample of patients diagnosed with depression, contributing to the limited literature on these constructs in psychiatric populations. Second, standardized measurement instruments with established Turkish validity and reliability were used in the study, and internal consistency coefficients obtained in the present sample were found to be acceptable. Third, the study evaluated both cognitive and forgiveness-related psychological processes together, providing a broader perspective on psychological functioning in patients with depression. Nevertheless, the findings should be interpreted within the methodological limitations of the study.

However, several limitations of the present study should be considered. First, the study was conducted in a single institution using a non-random sample without a healthy control group, which may limit the generalizability and comparability of the findings. Second, the data were obtained through self-report measures and may therefore be subject to response bias. Third, because of the cross-sectional design, causal or directional interpretations regarding the relationships among cognitive distortions, forgiveness of others, and self-forgiveness cannot be made. In addition, depression severity was not directly evaluated using a standardized symptom severity scale, and the findings should therefore be interpreted independently from symptom severity levels. Although participant diagnoses were verified through hospital medical records according to ICD-10 diagnostic criteria, possible unmeasured confounding variables may still have influenced the findings. Furthermore, psychiatric comorbidities, antidepressant use, psychotherapy history, treatment duration, current treatment status, illness duration, and recurrence characteristics were not specifically assessed and therefore were not included in the analyses. These unmeasured clinical and treatment-related factors may have influenced cognitive distortions, forgiveness of others, and self-forgiveness. Finally, the findings are limited to the instruments used and the characteristics of the study sample.

## 5. Conclusions

In conclusion, this study found that cognitive distortions were moderately and negatively associated with forgiveness of others, whereas a weak positive relationship was identified between forgiveness of others and self-forgiveness among patients with depression. In contrast, no statistically significant relationship was found between cognitive distortions and self-forgiveness. In addition, female participants were found to have higher self-forgiveness scores than males, and lower educational status was associated with higher cognitive distortion scores. These findings suggest that forgiveness-related processes and cognitive distortions may represent partially distinct psychological constructs in patients with depression. The nonsignificant relationship between cognitive distortions and self-forgiveness further suggests that self-forgiveness may be influenced by additional emotional, interpersonal, or contextual factors beyond cognitive distortions alone. Because of the cross-sectional design, causal or directional interpretations cannot be made. Future longitudinal and intervention-based studies are needed to better clarify the relationships among cognitive distortions, forgiveness, and self-forgiveness in clinical populations with depression.

In line with the findings of the present study, it may be beneficial for mental health professionals working with patients with depression to evaluate forgiveness-related processes and cognitive distortions together within psychosocial assessment and therapeutic approaches. Psychoeducational and therapeutic interventions targeting cognitive distortions may help address maladaptive thinking patterns associated with psychological functioning in depression. In addition, because no statistically significant relationship was found between cognitive distortions and self-forgiveness, self-forgiveness should be considered within broader emotional, interpersonal, and contextual frameworks rather than solely cognitive processes. The gender- and education-related differences identified in the study also suggest that sociodemographic characteristics should be considered when planning psychosocial support interventions. Furthermore, future studies using longitudinal, experimental, and intervention-based designs are recommended to better clarify the relationships among cognitive distortions, forgiveness, and self-forgiveness in patients with depression.

## Figures and Tables

**Table 1:** Descriptive characteristics of patients.

Descriptive Characteristics	Groups	n	%
Age (Mean = 36.57 ± 12.59; Min = 18, Max = 80)	≤30 years	91	36.1
	31–40 years	81	32.1
	41–50 years	45	17.9
	>50 years	35	13.9
Gender	Female	123	48.8
	Male	129	51.2
Marital status	Married	91	36.1
	Single	120	47.6
	Divorced	41	16.3
Educational status	Primary school	30	11.9
	Secondary school	18	7.1
	High school	83	32.9
	University	121	48.0
Employment status	Employed	153	60.7
	Unemployed	99	39.3
Diagnosis (ICD-10) *	F32 (Depressive episode, unspecified subtype)	53	21.0
	F32.0 (Mild depressive episode)	169	67.1
	F32.1 (Moderate depressive episode)	30	11.9
Total		252	100

Note: * ICD-10: International Statistical Classification of Diseases, 10th Revision; Min: Minimum; Max: Maximum.

**Table 2:** Mean total scores of Rye Forgiveness Scale, State Self-Forgiveness Scale and Cognitive Distortions Scale.

Scale (n = 252)	Possible Range	Observed Range	Mean ± SD
Rye Forgiveness Scale	15–75	21–58	39.78 ± 7.08
State Self-Forgiveness Scale	12–48	17–48	34.20 ± 7.71
Cognitive Distortions Scale	20–140	26–140	78.54 ± 20.98

Notes: SD: Standard Deviation.

**Table 3:** Differences between descriptive characteristics and scale total scores.

Descriptive Characteristics	Rye Forgiveness Scale Mean ± SD	State Self-Forgiveness Scale Mean ± SD	Cognitive Distortions Scale Mean ± SD
Gender			
Male	41.02 ± 7.36	34.96 ± 7.59	79.67 ± 21.76
Female	42.51 ± 6.75	37.38 ± 7.66	77.47 ± 20.24
	t = −1.672, *p* = 0.096	t = −2.519, *p* = 0.012	t = 0.829, *p* = 0.408
Marital Status			
Married (a)	43.14 ± 6.94	35.70 ± 8.15	74.69 ± 21.32
Single (b)	41.62 ± 7.10	36.64 ± 7.37	79.86 ± 21.03
Divorced (c)	39.27 ± 6.75	36.00 ± 7.75	83.24 ± 19.08
	F = 4.412, *p* = 0.013 post-hoc: a > c	F = 0.398, *p* = 0.672	F = 2.838, *p* = 0.060
Educational Status			
Primary School (a)	40.27 ± 7.44	34.03 ± 7.21	88.50 ± 16.09
Secondary School (b)	43.83 ± 5.60	38.11 ± 7.68	76.44 ± 19.35
High School (c)	40.39 ± 6.74	35.93 ± 6.86	79.77 ± 19.57
University (d)	42.82 ± 7.24	36.64 ± 8.32	75.55 ± 22.54
	F = 2.969, *p* = 0.133	F = 1.329, *p* = 0.265	F = 3.319, *p* = 0.021 post-hoc: a > c, a > d
Diagnosis (ICD-10) *			
F32 (Depressive episode, unspecified subtype)	41.51 ± 6.20	36.81 ± 7.17	79.62 ± 17.88
F32.0 (Mild depressive episode)	41.87 ± 7.35	35.81 ± 7.93	78.34 ± 22.20
F32.1 (Moderate depressive episode)	41.80 ± 7.19 F = 0.052, *p* = 0.949	37.33 ± 7.35 F = 0.712, *p* = 0.492	77.80 ± 19.40 F = 0.096, *p* = 0.908

Notes: SD = Standard Deviation; F = One-way ANOVA; t = Independent Samples *t*-test; post-hoc = Tukey HSD; *p* < 0.05; * ICD-10: International Statistical Classification of Diseases, 10th Revision.

**Table 4:** Correlations among Rye Forgiveness Scale, State Self-Forgiveness Scale, and Cognitive Distortions Scale scores.

Scales	1	2	3
1. Rye Forgiveness Scale (RFS)	1		
2. State Self-Forgiveness Scale (SSFS)	0.206 *	1	
3. Cognitive Distortions Scale (CDS)	−0.486 *	−0.039	1

Notes: * *p* < 0.01 (two-tailed). The correlation between CDS and SSFS was not statistically significant.

**Table 5:** Linear regression analyses examining the effects of cognitive distortions on forgiveness and self-forgiveness.

Predictor	Model 1: Forgiveness β	*p*	Model 2: Self-Forgiveness β	*p*
Cognitive Distortions Total	−0.486	<0.001	−0.039	0.533
Adjusted R^2^	0.233	—	−0.002	—
F	77.243	<0.001	0.390	0.533

Note: β = standardized regression coefficient. Model 1 dependent variable = Forgiveness Total; Model 2 dependent variable = Self-Forgiveness Total.

## Data Availability

The data presented in this study is available on request from the corresponding author. The data is not publicly available due to privacy and ethical restrictions, as it contains sensitive personal and health-related information.

## Abbreviations

The following abbreviations are used in this manuscript:

RFS	Rye Forgiveness Scale
SSFS	State Self-Forgiveness Scale
CDS	Cognitive Distortions Scale
WHO	World Health Organization
ICD-10	International Statistical Classification of Diseases, 10th Revision
